# The Polyphenolic Composition of Extracts Derived from Different Greek Extra Virgin Olive Oils Is Correlated with Their Antioxidant Potency

**DOI:** 10.1155/2019/1870965

**Published:** 2019-03-20

**Authors:** Paraskevi Kouka, Grigoria Tsakiri, Dimitra Tzortzi, Sofia Dimopoulou, Georgia Sarikaki, Panagiotis Stathopoulos, Aristidis S. Veskoukis, Maria Halabalaki, Alexios-Leandros Skaltsounis, Demetrios Kouretas

**Affiliations:** ^1^Department of Biochemistry and Biotechnology, University of Thessaly, 41500 Larissa, Greece; ^2^Department of Pharmacognosy and Natural Products Chemistry, Faculty of Pharmacy, University of Athens, 15771 Athens, Greece

## Abstract

Olive oil possesses a predominant role in the diet of countries around the Mediterranean basin, whereas it is a known constituent of several sectors of human culture. The polyphenolic composition of olive oil seems to be a key factor in its beneficial biological properties. Based on the above, the aim of this study was to correlate the polyphenolic composition of five extracts derived from a Greek olive oil variety with their antioxidant potency and antimutagenic activities *in vitro* with chemical-based techniques and cell culture-based assays. According to the results obtained, the polyphenol samples with higher concentration of hydroxytyrosol (HT) were more potent in antioxidant and antimutagenic activity *in vitro*, as indicated by their ability to scavenge ABTS^·+^ radical and to protect the strand of plasmid DNA from free radical-induced breaking compared to the corresponding samples with higher levels of tyrosol (T) and its derivatives. However, this observation was not evident in the cell culture model (i.e., the HeLa cervical cancer cell line) to which the tested extracts were administered. Specifically, the T-rich extracts more effectively increased endogenous GSH levels measured by flow cytometry than did the HT-rich compounds. Also, olive oil compounds contributed variously to the expression of genes implicated in the cell antioxidant machinery, as indicated by quantitative PCR. Therefore, the relationship between structure and function in redox regulation is complex and merits the combination of tests. Given that factors like the production and storage regimen of the plants are major determinants of the composition of the generated extracts, we propose that specific conditions should be adopted in order to achieve their maximum biological activity. These results followed by others in the same direction could provide a solid basis for the production of functional foods enriched in olive oil extracts with potential antioxidant action *in vivo*.

## 1. Introduction

Olive oil (OO), according to historical evidence, dates back to 6,000 BC. It was spread from Iran, Syria, and Palestine to the rest of the Mediterranean basin; thus, olive is one of the oldest known cultivated trees worldwide. Regarding Greece, it firstly appeared in Crete around 3,000 BC. In terms of symbolism, the olive tree is very important for Greeks as it is a component of their diet and their religious ceremonies [1]. The OO industry is one of the most important pillars of the Greek agrifood sector, and olive oil is a major product to be exported. Nowadays, Greece is the third largest olive oil producer worldwide (with Spain and Italy being the first and second, respectively), and the 70% of the total Greek OO production consists of extra virgin olive oil [2]. It is commonly used in cooking, cosmetics, pharmaceuticals, and religious customs and as fuel for traditional oil lamps.

Additionally, numerous studies have reported its great beneficial health effects, which are mainly attributed to its constituents, specifically the monounsaturated fatty acids (and mainly oleic acid), nutrients, vitamins, minerals, and, especially, polyphenols [3, 4]. Polyphenols are natural compounds of the secondary plant metabolism with a range of different chemical structures [5]. Plant polyphenols exert a wide spectrum of health-promoting properties such as anticancer, anti-inflammatory, antiallergic, antiatherogenic, and antimutagenic action. It is noteworthy that the biological activity of polyphenols is mostly related to their antioxidant potential due to their ability to neutralize reactive oxygen species (ROS) [3]. Taking the fact that the neurodegenerative diseases have a redox-related base into account, the nutritional antioxidants may play a crucial role in their prevention or alleviation. To this end, recent evidence points out the potential neuroprotective role of OO polyphenols, especially HT and oleocanthal, particularly against Alzheimer's disease [6].

The OO polyphenolic composition depends on several factors, such as the geographical zone, the agroclimatic conditions, the fruit ripeness, the cultivars, the storage conditions, and the oil extraction technology [7]. Specifically, the genetic variations among cultivars seem to affect the amount of polyphenols present in OO and, also, the polyphenols transferred from olive fruits to the produced oil. Therefore, polyphenolic compounds display both qualitative and quantitative variations among the cultivars, olive fruits, and olive oils [7, 8]. Moreover, the composition of OO polyphenols is altered during fruit processing, such as pressing and malaxation steps, due to the activity of enzymes or to the breakdown of compounds that exist originally in olive fruits. Thus, aglycone forms of oleuropein and ligstroside such as oleacein and oleocanthal as well as hydroxytyrosol (HT) and tyrosol (T) increase during the OO production [9]. Consequently, diverse technological processes have different impacts on the OO polyphenol content [4, 10]. Notably, it has been demonstrated that only 0.3%-1.5% of polyphenols are transferred from fruits to olive oil, while the 40% ends up in olive mill waste water [7].

In the oil extraction process, polyphenols are distributed between the oil and aqueous phases [11]. According to the amphiphilic nature of OO polyphenolic compounds, they exhibit greater solubility in the aqueous phase compared to the oily phase [12]. Considering the fact that each cultivar can absorb water in a different manner, the influence of the genetic background on the transfer of polyphenols from olive fruit to oil is considered a key factor. Thus, the magnitude of fruit moisture of each cultivar negatively affects the transfer of polyphenolic compounds to the oil [7]. Moreover, polyphenolic compounds, such as secoiridoids (e.g., oleuropein and ligstroside) as well as their aglycone forms, phenylalcohols (e.g., T and HT), flavonoids, and phenylethanoid glycoside (e.g., verbascoside and lignans), vary significantly among the cultivars and are altered during fruit development and maturation [13]. It is important to highlight that olive oil polyphenols are characterized by the presence of hydroxytyrosol (HT) and tyrosol (T). However, in most cases, the higher amounts are the derivatives thereof such as oleocanthal, oleacein, and aglycones of oleuropein and ligstroside in different forms. Specifically, they are compounds which contain in their basic structure T and HT moieties. Thus, the total amount of these compounds could be determined as equivalents after hydrolysis.

The most beneficial biological effects from OO are mostly attributed to the antioxidant activity of polyphenols. The main phenolic compounds identified in OOs are HT, T, secoiridoids, flavonoids, and lignans [14]. It has been observed that HT displays its chemopreventive role in tumor cell lines through generation and accumulation of hydrogen peroxide in the cell culture medium [15]. However, it is worth mentioning that when virgin oil phenolic extracts are tested in a complex mixture, they exert more potent chemopreventive action compared to the pure compounds probably due to their ability to act synergistically [15]. Furthermore, OO polyphenols possess the ability to scavenge both reactive oxygen (ROS) and nitrogen (RNS) species and, also, to protect DNA against oxidative modification. ROS and RNS can lead to the formation of highly promutagenic DNA adducts [16]. In addition, OO polyphenols have been shown to exert anticancer properties *in vitro* in different cell lines [14, 17].

Tyrosol and its metabolites can also ameliorate oxidative stress through scavenging of ROS, recycling of glutathione, and downregulation of glutathione peroxidase 1, glutamatecysteine ligase catalytic subunit, and heme oxygenase-1 genes [18]. Moreover, *in vivo* studies in animal models and humans have reported that HT, T, and their secoiridoid derivatives oleuropein aglycone, ligstroside aglycone, oleocanthal, and oleuropein inhibit the process of carcinogenesis [19] and reduce inflammatory-related pathologies, such as neurodegenerative diseases [20].

Based on the above, the aim of the present study was to examine the antioxidant and antimutagenic effects of five OO polyphenol extracts with substantial differences in their composition regarding hydroxytyrosol (HT), tyrosol (T), and their derivatives which are olive oil polyphenols with biological activity of utmost importance. Furthermore, we intended to make a holistic *in vitro* approach in order to obtain evidence that will be useful in relevant subsequent *in vivo* experiments with an ultimate goal to contribute to the production of potential biofunctional foods rich in olive oil polyphenols.

## 2. Materials and Methods

### 2.1. Chemicals, Reagents, and Culture Medium

Dulbecco's modified Eagle's medium (DMEM), fetal bovine serum (FBS), phosphate-buffered saline (PBS), 2,7-dichlorofluorescein diacetate (DCF-DA), mercury orange, and trypsin were purchased from Gibco (UK). Cell proliferation kit II (XTT) was purchased from Roche Diagnostics (Mannheim, Germany). Ethanol (EtOH) was purchased from Carlo Erba Reactifs SDS (Val de Reuil, France). Methanol (MeOH) was obtained from Fisher Scientific UK (Leicestershire, UK). All solvents were of analytical grade. Deionized water was used to prepare all aqueous solutions.

Methanol (MeOH), acetonitrile (ACN), H_2_O, and n-hexane were purchased from Macron Fine Chemicals (HPLC grade), acetic acid (CH_3_COOH) (laboratory reagent grade) was purchased from Fisher Scientific, and sulfuric acid (H_2_SO_4_) (for analysis) and 2,2′-azobis(2-methyl-propionamide) dihydrochloride (AAPH) were purchased from Sigma-Aldrich.

Virgin olive oil (VOO) samples obtained from olive (*Olea europaea L.*, cvs. Koroneiki variety), grown in Greece, coded as OLE05, OLE17, OLE19, OLE20, and OLE50, were chosen among a sample collection of the laboratory based on their phenolic composition. The selection included olive oils from olive (*Olea europaea L.*, Koroneiki variety), grown in Greece, collected in the harvesting period 2016/2017 with the following geographical origin, production system, and cultivation practice, respectively: OLE05 (Heraklion, Crete; two-phase mill; organic), OLE17 (Lakonia, Peloponnese; two-phase mill; integrated), OLE19 (Lakonia, Peloponnese; two-phase mill; integrated), OLE20 (Lakonia, Peloponnese; two-phase mill; integrated), and OLE50 (Lasithi, Crete; three-phase mill; conventional).

### 2.2. HPLC-DAD Analysis

The Thermo HPLC System consisted of a P4000 pump, an AS3000 autosampler, and a PDA detector. A Discovery HS C18 analytical column (15 cm × 4.6 mm, 5 *μ*m) was used. The solvent systems were water (0.2% AA)/acetonitrile (elution system A) and water/acetonitrile (elution system B). A gradient elution method of 20 min was applied, specifically from 98%A to 70%A for 17 min and back to initial conditions for 3 min. The low rate was sent in 1 ml/min.

### 2.3. Extraction Procedure

The selected OOs were subjected to extraction in order to obtain the polyphenol extracts. Specifically, liquid-liquid extraction according to the proposed method of IOC [21], with some modifications for the acceleration and automation of the procedure, was followed. Briefly, 1 gram of virgin OO was mixed thoroughly with 1 ml of n-hexane followed by the addition of 2 ml MeOH/H_2_O (8/2). The mixture was vortexed for 3 min and centrifuged at 3000 rpm for 3 min to separate the two phases. The organic phase was collected in another centrifuge tube, and the procedure was repeated twice. The combined organic phases containing the polar constituents of OO (total phenolic fraction (TPF)) were produced (TPF05, TPF17, TPF19, TPF20, and TPF50). All derived extracts were forwarded to acidic hydrolysis.

### 2.4. Determination of OO Polyphenols

In a next step, all the TPFs were hydrolysed under acidic conditions according to Mastralexi et al. Briefly, using the hydrolysis method, the derivatives of HT and T are hydrolysed affording HT and T to allow their quantification [22].

Regarding the hydrolysis method, an aliquot (200 *μ*l) of MeOH-water solution (6 : 4 (*v*/*v*)) of polar fraction was treated with 1 M H_2_SO_4_ (200 *μ*l). The mixture was maintained in a water bath for 2 h and forwarded for analysis. The quantification analysis of the hydrolysed forms of HT and T was performed using the calibration curve method. The calibration curves were created using five different concentration levels for both analytes. In fresh olive oils, the levels of HT and T are extremely low (HT: 0.03-0.5; T: nd-0.43 mg/20 g oil) while their derivatives are in very high levels. Thus, after hydrolysis, the determined polyphenols are expressed as HT and T derivatives.

Taking the fact that the mean molecular weight (MW) of the 10 most known bound forms of HT and T is ~346 amu into consideration, a correction factor for both HT and T (HT: 2.2, T: 2.5) was introduced as described previously by Mastralexi et al. [22]. Thus, in the regression equation of HT (*y* = 87968*x* − 11437, *R*^2^ = 0.999) and T (*y* = 55018*x* − 29720, *R*^2^ = 0.999), the above correction factors were introduced (Table 1).

Taking the concentration levels of HT and T in the analysed TPF (Table 1) into account, it is obvious that the OOs under investigation are also rich in derivatives of HT and T. The most representative ones are secoiridoid aglycones. It should be noted that phenolic alcohols including HT and T appear in low concentrations in fresh olive oils, a phenomenon that is reversed during oil storage [23]. The latter can be formed by the hydrolysis of secoiridoids, such as oleacein (3,4-DHPEA-EDA), oleocanthal (pHPEA-EDA), isomers of oleuropein aglycone (3,4-DHPEA-EA), and aglycone derivatives of oleuropein and ligstroside (p-HPEA-EA) containing 3,4-DHPEA and p-HPEA in their molecular structures [24]. Moreover, these substances can also be generated during the oil mechanical extraction process after hydrolysis of oleuropein, demethyloleuropein, and ligstroside. This procedure is known to be catalyzed by endogenous beta-glucosidases [25]. Based on the results (Table 1), OLE20 and OLE50 could be considered equal in the concentration levels of HT and T derivatives, in contrast to OLE05 which is rich in T (T-rich) and OLE17 and OLE19 which are rich in HT (HT-rich).

### 2.5. ABTS^·+^ Radical Scavenging Assay

The ABTS^·+^ radical scavenging capacity (RSC) of the tested extracts was determined based on the protocol described by Cano et al. [26] with minor modifications [27]. Briefly, in a total reaction volume of 1 ml in distilled water (dH_2_O), ABTS^·+^ (1 mM), H_2_O_2_ (30 *μ*M), and horseradish peroxidase (HRP) (6 *μ*M) in 50 mM phosphate-buffered saline (pH = 7.5) were added. The solution was incubated for 45 min at room temperature (RT) in the dark. Finally, 10 *μ*l of the tested extracts at various concentrations was added, and the absorbance was monitored on a Hitachi U-1900 radio beam spectrophotometer (serial no. 2023-029; Hitachi Ltd.) at 730 nm. In each experiment, a blank without the HRP was used, while the ABTS^·+^ radical solution without the extract was used as the control. The RSC percentage of the tested extracts was calculated using the following equation: RSC (%) = [(OD_control_ − OD_sample_)/OD_control_] × 100, where OD_control_ and OD_sample_ are the optical density (OD) values of the control and the test sample, respectively. Moreover, the IC_50_ value indicating the amount of the extract that induced scavenging of the ABTS^·+^ radical at 50% was calculated. The experiments were conducted in triplicate (three repetitions) and also at 3 different independent times (separate occasions). Furthermore, as a reference substance, vitamin C was used, as indicated by Kim et al. [28]. In a research, where a range of different radical scavenging tests were used for the virgin OO examined, the results showed that the best method for determining the antioxidant capacity of OO was the ABTS^·+^ assay [29].

### 2.6. DNA Relaxation Assay

The DNA relaxation assay has been already described [11, 27]. The plasmid (pBluescript SK+, Fermentas, Waltham, MA, USA) DNA normally exists in the supercoiled conformation, but following a single-strand break, it is converted to the open circular conformation. Based on this principle, the protective activity of the olive oil extracts against DNA single-strand breaks by AAPH (2.5 mM) was assessed. Briefly, in a total reaction volume of 10 *μ*l, 2 *μ*l (4 *μ*g/ml) of DNA was mixed with PBS and a range of different concentrations of the tested olive oil extract. The tested concentrations ranged between 1 and 300 *μ*g of extract. The tubes were incubated for 45 min at 37°C. Finally, 3 *μ*l of loading buffer (containing bromophenol blue 0.25% + 30% glycerol) was mixed, and the samples were loaded on a 0.8% agarose gel. The samples were run at 70 V for 60 min. Subsequently, the gel was stained with 12.5 *μ*l of ethidium bromide (10 mg/ml) in 250 ml of dH_2_O for 30 min. Consequently, the gel was washed with 250 ml of dH_2_O for 30 min. Finally, the gels were exposed to UV, the MultiImage Light Cabinet (Alpha Innotech, San Leandro, CA, USA) was used to capture the gel photos, and the results were analyzed with the Alpha View suite. For negative control, DNA was mixed with PBS only, and for positive control, DNA was mixed with both PBS and AAPH. The maximum tested concentrations were mixed with DNA and PBS, without the AAPH, to check the putative effects of the extracts on plasmid DNA. None of the tested concentrations induced DNA breaks.

### 2.7. Cell Culture Conditions

The cervical cancer cells (HeLa) were cultured in Dulbecco's modified Eagle's medium (DMEM) containing 10% (*v*/*v*) fetal bovine serum (FBS), 2 mM of L-glutamine, 100 U/ml of penicillin, and 100 U/ml of streptomycin (all from Gibco, Paisley, UK) at 37°C in 5% CO_2_.

### 2.8. Cell Viability Assay

Cell viability was assessed using the XTT assay kit (Roche, Mannheim, Germany) as described previously [27]. Briefly, 1 × 10^4^ HeLa cells per well were cultured in a 96-well plate in DMEM. Following a 24 h incubation, a wide range of olive oil extract concentrations diluted in a serum-free DMEM were administered for 24 h. Subsequently, 50 *μ*l of the XTT reagent (50 : 1) was added to each well. After 4 h of incubation, the absorbance was monitored at 450 nm and also at 630 nm as a reference wavelength in a BioTek ELx800 microplate reader (BioTek Instruments Inc., Winooski, VT, USA). As a negative control, samples containing serum-free DMEM only were used. In addition, the absorbance of the extracts alone in serum-free DMEM and XTT test solution was measured at 450 nm and 630 nm. The absorbance values of the extracts alone were subtracted from those derived from the absorbance of the cells treated with the tested compounds. Data were calculated as the percentage of viability using the following equation: viability (%) = [(OD_control_ − OD_sample_)/OD_control_] × 100, where OD_control_ and OD_sample_ indicate the OD of the negative control and the tested compounds, respectively. The experiments were conducted in triplicate (three repetitions) and also at 3 different independent times (separate occasions).

### 2.9. Measurement of Endogenous GSH and ROS Levels in the HeLa Cell Line Using Flow Cytometry

The intracellular GSH and ROS levels were assessed using the fluorescent dyes, mercury orange, and 2,7-dichlorofluorescein diacetate (DCF-DA), respectively [11, 27]. Mercury orange binds directly to GSH, while DCF-DA is deacetylated within cells by esterases and is further converted to fluorescent DCF by the oxidative action of ROS. A 400 *μ*M stock solution of mercury orange was prepared in acetone, and a 400 *μ*M stock solution of DCF-DA was prepared in methanol. Firstly, the cells were trypsinized and centrifuged (300 g, 5 min, 4°C). Afterwards, the cell pellet was resuspended in PBS at the concentration of 1 × 10^6^ cells/ml and incubated in the presence of mercury orange (40 *μ*Μ) or DCF-DA (10 *μ*Μ) at 37°C for 30 min. The cells were then washed and resuspended in PBS and subjected to flow cytometric analysis using a FACSCalibur flow cytometer (BD Biosciences, Franklin Lakes, NJ, USA) with excitation and emission wavelengths at 488 and 530 nm, respectively, for ROS and at 488 and 580 nm, respectively, for GSH. The cells were analyzed at a flow rate of 1,000 events/sec. Analyses were performed on 10,000 cells per sample, and the fluorescence intensities were measured on a logarithmic scale. Data were analyzed using BD Cell Quest software (BD Biosciences). Each experiment was repeated at least 3 times.

### 2.10. Quantitative PCR (qPCR)

RNA was extracted from the cell pellet using an RNA isolation kit (PureLink™ RNA kit; Invitrogen; Thermo Fisher Scientific Inc., Waltham, MA, USA). Afterwards, the extracted RNA (~10 *μ*g) was treated with DNase (RQ1 RNase-Free DNase, 1 U/*μ*l; Promega Corporation, Madison, WI, USA). DNA-free RNA was then reverse transcribed to obtain cDNA (Superscript II Reverse Transcriptase) using oligo (dT) 12-18 primers (both from Invitrogen; Thermo Fisher Scientific Inc.). Amplification of cDNAs for the Nrf2 target genes (cat, sod1, txn, hmox1, nrf2, nqo1, gclc, gsr, and gpx1) and for the gapdh gene was performed in a total volume reactions of 10 *μ*l containing SYBR® Select Master Mix (2X; Applied Biosystems; Thermo Fisher Scientific Inc.), 0.25 *μ*Μ of each primer, 50 nM ROX Low, and 25 ng cDNA for the amplification of all the tested genes. The primer sequences are presented in Table 2. The thermocycling conditions used for the amplification of genes were 3 min at 95°C, 45 cycles of 15 sec at 95°C, and 30 sec at 53°C for all the genes. Finally, a melting curve was carried out from 53 to 95°C to check the specificity of the products. qPCR was performed on a MX3005P system (Stratagene, UK). Amplification efficiencies were >87% with *R*^2^values > 0.982 for the genes.

### 2.11. Statistical Analysis

All data were analyzed using one-way ANOVA followed by Dunnett's tests for multiple pairwise comparisons. Data are presented as mean ± SEM, and the significance level was set at *p* < 0.05. SPSS version 21.0 was used (SPSS Inc., Chicago, IL, USA).

## 3. Results

**Figure 1:**
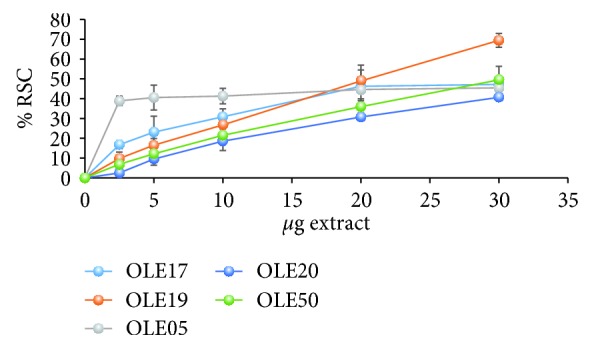
Antioxidant capacity of the tested OO extracts measured by their ability to reduce/scavenge ABTS^·+^ radical. OLE17 (*R*^2^ = 0.9026) and OLE19 (*R*^2^ = 0.9994) are HT-rich olive oils. OLE05 (*R*^2^ = 0.9423) is a T-rich olive oil, and OLE20 (*R*^2^ = 0.9747) and OLE50 (*R*^2^ = 0.9947) are olive oils with an equal amount of HT and T. The results are expressed as the means ± SEM (*p* < 0.05, *n* = 3). HT: hydroxytyrosol; T: tyrosol; RSC: radical scavenging capacity. *R*^2^: coefficient of determination.

As indicated by the results regarding the ABTS^·+^ assay, all extracts exhibited potent antioxidant activity, compared to vitamin C (reference substance) (Figure 1 and Table 3). Our reference substance was vitamin C, which according to Kim et al. [28] is widely used that way in order to assess the antioxidant activity of the desirable compounds. We observed that the IC_50_ of vitamin C is equal to 6.5 *μ*g/ml and the IC_50_ of our extracts ranged between 28.58 and 46.55 *μ*g/ml. Specifically, the IC_50_ values of OLE17, OLE19, OLE05, OLE20, and OLE50 were found to be equal to 29, 21, 47, 36, and 30 *μ*g of extract, respectively, while the IC_50_ value for vitamin C was equal to 6.5 *μ*g/ml. Given that our extracts are a mixture of compounds, unlike vitamin C, we conclude that they exert potent antioxidant activity. IC_50_ represents the concentration of the tested compounds required for 50% reduction of the ABTS^·+^ radical. The low value of IC_50_ indicates that an extract exhibits more powerful antioxidant activity. Thus, the results revealed that OLE17 and OLE19 had stronger antioxidant potency compared with OLE20, OLE50, and OLE05 with the latter being the weakest OO extract. It is noteworthy to be mentioned that our extracts are a mixture of compounds and not a pure compound, as vitamin C.

**Figure 2:**
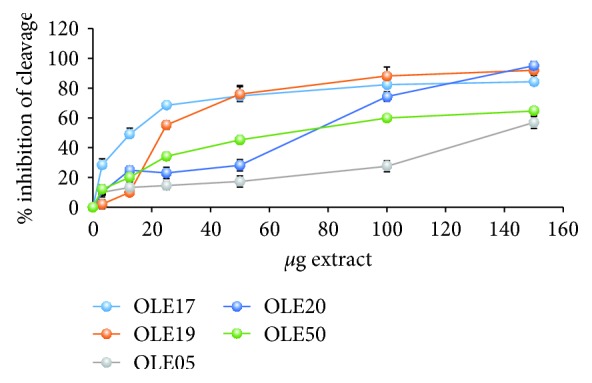
Antigenotoxic capacity of the tested OO extracts measured by their ability to protect DNA cleavage (i.e., single-strand breaks) induced by ROO^·^ radicals. OLE17 (*R*^2^ = 0.7501) and OLE19 (*R*^2^ = 0.7757) are HT-rich olive oils. OLE05 (*R*^2^ = 0.9349) is a T-rich olive oil, and OLE20 (*R*^2^ = 0.9379) and OLE50 (*R*^2^ = 0.8205) are olive oils with an equal amount of HT and T. All results are shown as the means ± SEM (*p* < 0.05, *n* = 3). HT: hydroxytyrosol; T: tyrosol; *R*^2^: coefficient of determination.

The results for the plasmid relaxation assay (Figure 2 and Table 3) revealed that OLE17 and OLE19 had lower IC_50_ values and, hence, higher antigenotoxic activity. Specifically, the IC_50_ values of OLE17 and OLE19 were calculated at 14 and 23 *μ*g of extract, respectively. Moreover, OLE20 and OLE50 were less potent with IC_50_ at 74 *μ*g and 66 *μ*g, respectively. Finally, OLE05 with IC_50_ at 133 *μ*g showed the lowest ability to protect DNA from the detrimental effects of ROO^·^ radicals.

The antioxidant activity of the tested compounds was examined in HeLa cells. The activity of polyphenols in HeLa plays a vital role, among the chemopreventive agents, for the prevention of cancer. Before considering the potential therapeutical applications of the olive oil extracts, it is crucial to demonstrate that it does not present cytotoxic effects in the tested cell line. Thus, the XTT assay (Table 4) was used in order to evaluate the noncytotoxic concentrations. According to the results in cell cultures, OLE19 and OLE17 exhibited cytotoxicity at concentrations > 30 *μ*g, OLE50 at 20 *μ*g, and OLE20 and OLE05 at 50 *μ*g of extract.

**Figure 3:**
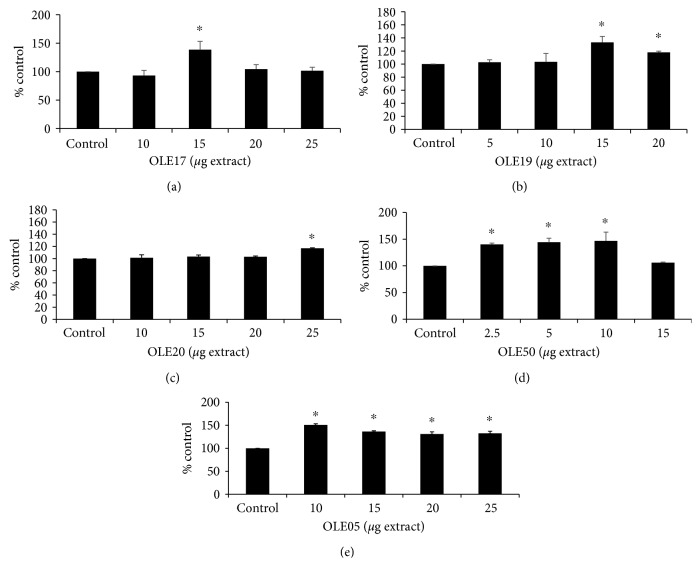
The effects of the tested OO extracts on GSH levels of HeLa cells after 24 h of treatment. (a) OLE17. (b) OLE19. (c) OLE20. (d) OLE50. (e) OLE05. Bar charts showing the GSH levels, as calculated by BD Cell Quest software. All results are expressed as the means ± SEM of 4 experiments (*n* = 4). ^∗^*p* < 0.05 indicated a statistically significant difference between OO extracts and the control. GSH: reduced form of glutathione.

The results obtained from flow cytometric analysis revealed that treatment of the HeLa cells with all the tested OO extracts significantly increased GSH levels compared with the control (Figure 3). Regarding the HT-rich OO, OLE17 (Figure 3(a)) increased GSH levels at 15 *μ*g of extract by 39% and OLE19 (Figure 3(b)) increased GSH levels by 33% and 18% at 15.0 and 20.0 *μ*g, respectively, compared with the control. Regarding the T-rich OO, OLE05 (Figure 3(e)) increased GSH levels by 51%, 36%, 31%, and 33% at 10.0, 15.0, 20.0, and 25.0 *μ*g, respectively, compared with the control. Finally, GSH levels were also found elevated after incubation with OO with an equal amount of HT and T. Specifically, OLE20 (Figure 3(c)) depicted an increase by 17% at 25.0 *μ*g and OLE50 (Figure 3(d)) by 40%, 44%, and 47% at 2.5, 5.0, and 10.0 *μ*g, respectively, compared with the control.

**Figure 4:**
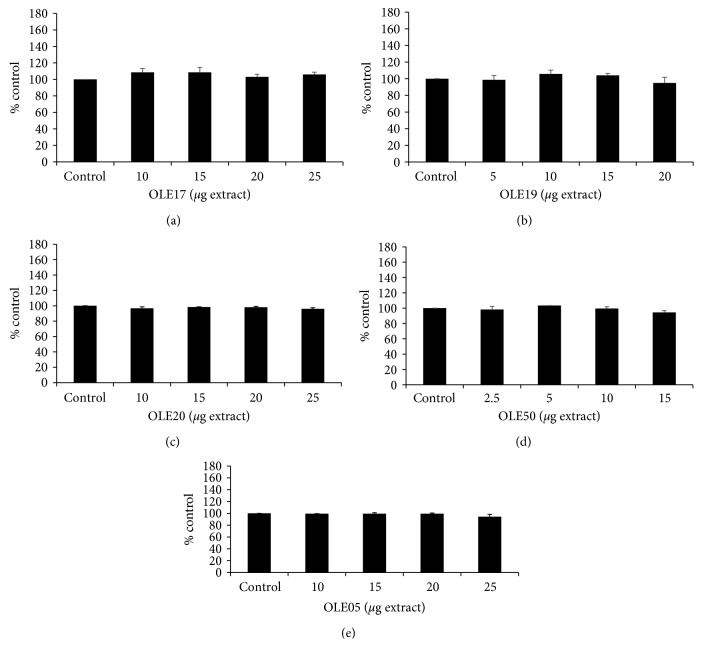
Effects of the tested compounds on ROS levels of HeLa cells after treatment for 24 h, as assessed by flow cytometry. (a) OLE17. (b) OLE19. (c) OLE20. (d) OLE50. (e) OLE05. Bar charts showing the ROS levels, as calculated by BD Cell Quest software. All results are expressed as the means ± SEM of 4 experiments (*n* = 4). ^∗^*p* < 0.05 indicated a statistically significant difference between OO extracts and the control. ROS: reactive oxygen species.

Unlike GSH, the ROS levels were not significantly affected by any of the tested OO compared with the controls (Figure 4). It should be mentioned that the measured ROS levels correspond to the naturally occurring levels in HeLa cells; that is, there was not any treatment of cells with oxidizing agents before the addition of OO extracts.

The OO extracts were selected for further analysis in order to shed light on olive oils' mechanism of action. Based on the literature, polyphenols are able to cause Nrf2 detachment from the cytosol-localized Keap1 and subsequent translocation to the nucleus followed by antioxidant gene expression [30, 31]. The OO extracts were administered in HeLa cells at concentrations that caused the maximum increase in GSH levels and sequentially were assessed for their effect on gene expression levels of Nrf2 target genes by qPCR. Specifically, OLE17 and OLE19 were administered at 15.0 *μ*g, OLE50 and OLE05 at 10.0 *μ*g, and OLE20 at 20.0 *μ*g. The tested genes encode for catalase (*cat*), superoxide dismutase 1 (*sod1*), thioredoxin (*txn*), heme oxygenase 1 (*hmox1*), Nrf2, NAD(P)H quinone dehydrogenase 1 (*nqo1*), *γ*-glutamyl cysteine ligase catalytic subunit (*gclc*), glutathione reductase (*gsr*), and glutathione peroxidase 1 (*gpx*). The expression of all the aforementioned genes is subject to regulation by an antioxidant response element (ARE) in their promoter region, recognized by the Nrf2. These proteins are a part of the complex antioxidant machinery that protects cells from oxidative impairments by neutralizing ROS [31]. Therefore, extracts or compounds that upregulate their expression may be used as potential antioxidant supplements.

**Figure 5:**
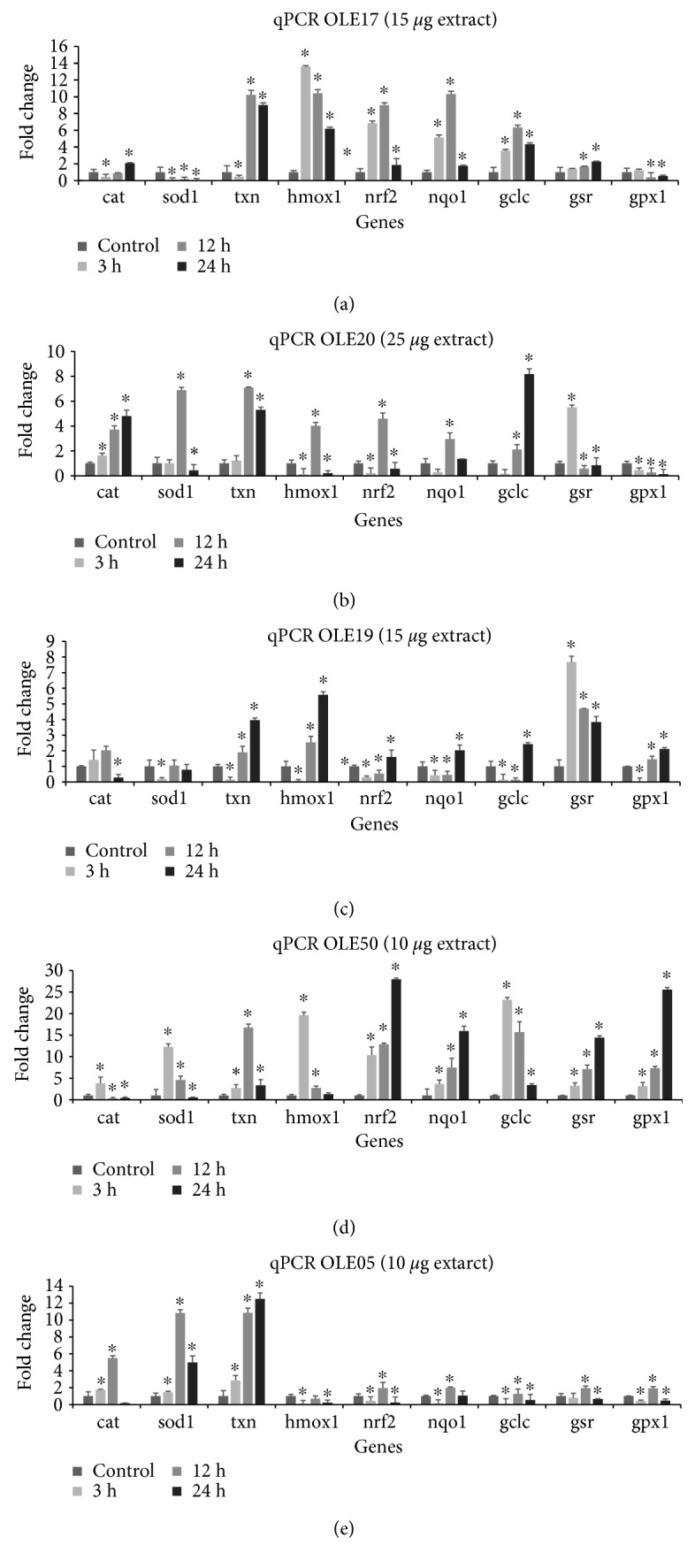
Effect of the tested OO extracts on Nrf2 target gene expression in HeLa cells following administration of extracts for 3, 12, and 24 h, using qPCR. Gene expression has been normalized using gapdh expression and the fold change in gene expression in comparison to the control cells. The results are expressed as mean ± SEM of 3 individual experiments (*n* = 3), ^∗^*p* < 0.05 indicated a statistically significant difference between OO extracts and the control. Nrf2: nuclear factor erythroid 2-related factor 2.

According to the qPCR results (Figure 5), all the OO extracts upregulated most of the tested genes compared to the control. We present the greater percentage of gene expression upregulation indicating the most potent biological effect. Specifically, OLE17 (Figure 5) caused the upregulation of *cat* by 2.06-fold at 24 h, *txn by* 10-fold at 12 h, *hmox1* by 13-fold at 3 h, *nrf2* by 9-fold at 12 h, *nqo1* by 10.3-fold at 12 h, *gclc* by 6.3-fold at 12 h, *gsr* by 12.3-fold at 24 h, and *gpx1* by 1.2-fold at 3 h. On the other hand, *sod1* and *gpx1* levels were decreased only at 12 h and 24 h. Following the qPCR results obtained after the OLE19 (Figure 5) administration, an upregulation of *cat* levels by 2-fold at 12 h, *sod1* by 1.04-fold at 12 h, *txn* by 5.6-fold at 24 h, *hmox1* by 5.6-fold at 24 h, *nrf2* by 1.6-fold at 24 h, *nqo1* by 2.02-fold at 24 h, *gclc* by 2.4-fold at 24 h, *gsr* by 7.7-fold at 3 h, and *gpx1* by 2.1-fold at 24 h was observed. On the contrary, the majority of the genes were found decreased mostly at 3 h. OLE20 administration (Figure 5) upregulated most of the genes compared to the control, specifically *cat* by 4.8-fold at 24 h, *sod1* by 6.9-fold at 12 h, *txn* by 7.1-fold at 12 h, *hmox1* by 4.5-fold at 12 h, *nrf2* by 2.9-fold at 12 h, *nqo1* by 2.9-fold at 24 h, *gclc* by 8.2-fold at 24 h, and *gsr* by 5.5-fold at 3 h, while a significant decrease was observed at the rest of the time points. Additionally, OLE50 administration (Figure 5) upregulated the expression of the following genes: *cat* by 3.8-fold at 3 h, *sod1* by 12.2-fold at 3 h, *txn* by 16.7-fold at 12 h, *hmox1* by 19.6-fold at 3 h, *nrf2* by 27.9-fold at 24 h, *nqo1* by 15.9-fold at 24 h, *gclc* by 23.2-fold at 3 h, *gsr* by 14.4-fold at 24 h, and *gpx1* by 25.5-fold at 24 h. Finally, OLE05 upregulated (Figure 5) *cat* by 5.5-fold at 12 h, *sod1* by 10.8-fold at 12 h, *txn* by 12.5-fold at 24 h, *nrf2* by 1.9-fold at 12 h, *nqo1* by 1.9-fold at 12 h, *gclc* by 1.3-fold at 12 h, *gsr* by 1.9-fold at 12 h, and *gpx1* by 1.9-fold at 12 h.

## 4. Discussion

In the present study, the potential antioxidant and antigenotoxic properties of 5 polyphenolic virgin OO extracts were investigated. The extracts varied in terms of their content in HT and T derivatives, which are the major polyphenolic compounds present in OO with numerous beneficial properties for human health. According to the obtained results, the HT-rich OO extracts were the strongest antioxidant and antigenotoxic agents *in vitro*. On the contrary, the T-rich OO extract was the most effective in enhancing cell GSH levels, whereas none of the tested compounds had any effect on cell ROS levels. It is worth mentioning that HT and T are released after oleocanthal and oleacein hydrolysis, respectively, by endogenous enzymes during ripening of olive drupe and malaxation [32].

To evaluate the antioxidant capacity of the olive oil (OO) extracts, we used two different models, namely, chemical-based and cell culture-based techniques. Although cell cultures are considered an *in vitro* system, due to the fact that they are metabolically active, they are considered an *in vivo*-like environment, thus providing a link to *in vivo* settings [23]. The ABTS^·+^ assay is a useful tool to detect the antioxidant capacity of OO polyphenols. Moreover, we used the DNA relaxation assay because it allows the revelation of natural molecules potentially able to protect DNA from the damaging effects of various oxidants. It is known that DNA is more prone to oxidative modifications compared with other biomolecules; therefore, this assay is considered suitable for the assessment of the protective effect of naturally occurring antioxidants [24]. The HeLa cells are a commonly used model in order to evaluate the effects of plant extracts and other similar compounds on cell redox status.

According to the ability of the extracts to scavenge ABTS^·+^ radical, all the OO extracts had a great antioxidant activity. However, the HT-rich OO extracts seem to be more potent antioxidants compared to the T-rich ones, whereas compounds with common structures exhibited similar antioxidant activity. It is worth mentioning that the antioxidant activity of a polyphenolic compound is usually enhanced when the number of the phenolic groups present in the molecule is increased [33]. Specifically, molecules with *o*-dihydroxyl moieties possess high antioxidant capacity through the formation of hydrogen bonds with free radicals. Moreover, an electron-donating substance in “ortho” position seems to weaken the O-H bond of the polyphenol, thus providing greater stability to the phenoxyl radical [34]. Therefore, when an antioxidant reacts with a free radical, it provides an electron and becomes a weak, nontoxic free radical itself [29]. To this end, HT exhibits higher antioxidant capacity compared to T indicating that the higher antioxidant capacity of HT-rich OO extracts may be due to the higher number of hydroxyl groups of the HT molecule.

The antiradical potential of the OO extracts has been thoroughly examined. Specifically, in a study conducted from our research group [27], an OO polyphenolic fraction had the ability to scavenge *in vitro* both the ABTS^·+^ and DPPH^·^ radicals. Moreover, it has been reported that the DPPH^·^ radical-scavenging activity of extra virgin olive oil (EVOO) correlates with its polyphenolic content while OO extracts through its polyphenols display potent antiradical action [35]. A relevant study has shown that the olive fruit maturity levels decrease the polyphenolic content of OO extracts and consequently their efficiency [36]. Thus, it is important to control the olive ripening level in order to achieve the optimum harvesting time [36].

Similar to their antioxidant potential, the OO extracts depicted the same pattern in the DNA relaxation assay. Specifically, OLE17 and OLE19 (i.e., the OO extracts with higher amounts of HT) exhibited lower IC_50_ values; hence, they have the strongest antigenotoxic activity among all the tested samples. The aforementioned data is in agreement with a study conducted on human mammary epithelial cells (MCF10A), according to which HT has a greater protective effect on DNA oxidation than T [37]. Also, HT exhibited significantly higher protective capacity against hydrogen peroxide-induced DNA damage compared to T in human blood mononuclear cells (PBMC) and promyelocytic leukemic cells (HL60) [38]. Moreover, it has been demonstrated that HT, but not T, which lacks the hydroxyl group at position 3 is able to protect Jurkat cells, an immortalized line of human T lymphocyte cells, from hydrogen peroxide-induced DNA damage indicating that the presence of aromatic rings with orthodihydroxy moieties is required for enhanced protective activity [39].

After 24 hours of incubation of HeLa cells with the OO extracts, GSH levels were measured using flow cytometry. GSH is one of the most important intracellular antioxidant molecules that protect cells from oxidative damage. GSH plays a critical role in signaling pathways, in detoxification processes of certain antibiotics and heavy metals, in the antioxidant system, and in metabolic pathways of most aerobic cells due to its reducing properties [40, 41]. According to our results, HT-rich OO extracts exhibited the maximum increase in GSH levels at 15 *μ*g of extract in a range of 35-40%, compared to the control. On the other hand, T-rich OO extracts depicted the maximum increase in GSH levels at 10 *μ*g by 50%. Also, at 15 *μ*g of Τ-rich OO, GSH was increased about 40%. Moreover, OLE17 had no effect at 20 *μ*g on GSH levels, and OLE19 increased GSH levels by 18% compared with control while OLE05 by 31%. Many studies have reported that both HT and T upregulate the Keap1/Nrf2 pathway, which is responsible for the expression of the enzymes involved in the GSH synthesis, such as *γ*-GCL [42–45]. Experiments in rats have identified T as a precursor of HT suggesting that the initial composition of the extracts administered could have been altered after the conversion of a molecule to other metabolites [46]. Moreover, T was found to be converted into HT in human liver microsomes (HLM) through the CYP2A6 and CYP2D6 enzymatic activities [47]. It has also been reported that in J774 A.1 cells (mouse BALB/c monocyte macrophage), T has the ability to accumulate intracellularly over time until it reaches high concentrations and, hence, it exhibits significant protective and antioxidant effects [48]. Unlike T, the levels of HT in the cells decreased very quickly [48]. Furthermore, when HT was administered in Jurkat cells at several time points, a protective effect was observed after 5 min of incubation, which was gradually decreased as the incubation time was extended up to 2 h. These findings indicate probably the metabolic inactivation of HT [39]. Therefore, cellular metabolism seems to significantly affect the activity of extracts rich in polyphenols. It should be mentioned that HeLa cells, as cancer cells, are highly metabolically active. Regarding ROS levels, unlike GSH, they remained unaltered. Previous studies have also reported that changes in oxidative stress levels or antioxidant mechanisms are not always accompanied by changes in ROS levels [27, 49, 50].

The ability of phenolic compounds to interact with biological system monitoring gene expression (e.g., Nrf2, nuclear factor-*κ*B, MAP kinase, and PI3 kinase/Akt) is one of the mechanisms that partly explain their health contributions [30]. A wide spectrum of plant extracts has been examined in both *in vivo* and *in vitro* models to assess different phenolic compounds as stimulators of the Nrf2 pathway [30, 51]. In the present study, we focus on phenolic compounds from OO and their ability to interact with Nrf2, which plays a predominant role in regulating the expression of genes implicated in the cell antioxidant defense system. According to our results, all the tested OO extracts had the ability to upregulate the genes encoded by Nrf2 with the greatest increase in the majority of the genes, to be observed at 12 h of administration. The main OO phenolic compounds, namely, HT, T, and oleuropein, can exert their beneficial effects by stimulating the Nrf2 pathway, thus eliminating oxidative stress moieties, such as ROS and RNS [30].

However, the aforementioned applications describe only the *in vitro* antioxidant potential of plant compounds. Considering the intricacy of oxidation processes, there is no sole-assay approach that totally describes the antioxidant profile of a substance. Thus, the combination of different methods and systems, in order to holistically reveal an extract's antioxidant capacity in the frame of redox biology, seems to be mandatory. Towards this direction, it is crucial to demonstrate the effects of an extract on a cell culture environment also, both in physiological and cancer cell lines. Polyphenols are metabolized in a cell environment [52]. Therefore, polyphenolic extracts combined with their metabolites may contribute to the reinforcement of the antioxidant environment, thus preserving cell integrity and function.

Our results point out the necessity of the *in vitro* and the cell culture system interference. Specifically, the OO extracts had the ability to detoxify the ABTS^·+^ radical *in vitro*, although there are no statistical alterations at ROS levels in the tested cell line. In the *in vitro* assay, the antioxidant compounds interact directly with the ABTS^·+^ radical, but in the cellular environment, which is a living system, there are a lot of different reactive species, such as hydrogen peroxide, hydroxyl radicals, superoxide anion, and peroxynitrite [53]. Also, the extract reductive potential differs between the two models. The OO extracts had the ability to donate an electron and thus to detoxify the ABTS^·+^ radical in different concentrations compared with their ability to increase GSH levels in cells. Specifically, the OO concentrations in which the IC_50_ is achieved in the chemical-based assays are toxic in the tested cell line, and thus, GSH levels increased at lower concentrations.

About 80-90% of the total VOO polyphenolic content exists in the form of secoiridoids. In the gastric environment, secoiridoids are hydrolysed; thus, free polyphenols are available for absorption in a dose-dependent manner. Therefore, polyphenols, such as HT and T, undergo an extensive first-pass metabolism. Consequently, the concentration of HT and T in biological fluids may be different compared to their metabolites, which may exert a different biological role [18].

Moreover, in the present study, we evaluated the antioxidant potential of the total polyphenolic fraction from the different OO extracts. In another study, a comparison between the antioxidant effects of both the total polyphenolic extract and some of their single purified phenols revealed that the total phenol extract possessed a stronger antioxidant capacity compared with each compound separately. This points out the synergistic or accessory effect of the multiple compounds compared to the single purified ones [54].

## 5. Conclusions

The strong points of this study can be summarized in the fact that the antioxidant and antigenotoxic activities of the total OO extracts, and not only the action of pure compounds, have been evaluated providing a more holistic aspect about their potency [55]. Furthermore, the biological activity of the tested extracts has been evaluated using a combination of chemical-based assays and cell models to evaluate their potential biological role. Nevertheless, the limited number of extracts could be a restriction point for the full evaluation of OO biological effects.

To conclude, our results indicate that all the five tested OO extracts exhibited potent antioxidant activity, as well as protective action against free radical-induced DNA damage assessed by *in vitro* assays. In particular, our findings point out that the HT-rich extracts are stronger antioxidant and antigenotoxic agents compared with the T-rich compounds. This, however, was not the case in the cell-based model (i.e., HeLa cells) since T-rich extracts were more effective in increasing GSH levels compared to the HT-rich compounds, highlighting that cell metabolism is able to alter the activity of any compound administered. Given that factors like the production and storage regimen of the plants are major determinants of the composition of the generated extracts, we propose that specific conditions should be adopted in order to achieve their maximum biological activity. These results followed by others in the same direction could provide a solid basis for the production of functional foods enriched with OO extracts, which will exhibit potent antioxidant action *in vivo*.

## Figures and Tables

**Table 1:** Concentrations (mg/g OO) of hydroxytyrosol and tyrosol derivatives (bound forms).

Sample	mg HT derivatives/g of OO	mg T derivatives/g of OO
OLE05	0.095	0.270
OLE17	0.257	0.165
OLE19	0.282	0.217
OLE20	0.156	0.177
OLE50	0.119	0.140

**Table 2:** Primer sequences.

Gene	Access no.	Primer (5′-3′) human
*cat*	847	Forward: CCAGAAGAAAGCGGTCAAGAA
		Reverse: TGGATGTGGCTCCCGTAGTC
*sod1*	6647	Forward: AGGGCATCA TCAATTTCGAG
		Reverse: GGGCCTCAGACTACATCCAA
*txn*	7295	Forward: TTTCCATCGGTCCTTACAGC
		Reverse: TTGGCTCCAGAAAATTCACC
*hmox1*	3162	Forward: GGCCTGGCCTTCTTCACCTT
		Reverse: GAGGGGCTCTGGTCCTTGGT
*nrf2*	4780	Forward: *ATTGCCTGTAAGTCCTGGTCA*
		Reverse: *ACTGCTCTTTGGACATCATTTCG*
*nqo1*	1728	Forward: GGGCAAGTCCATCCCAACTG
		Reverse: GCAAGTCAGGGAAGCCTGGA
*gclc*	2729	Forward: GAAGAAGATATTTTTCCTGTCATTGAT
		Reverse: CCATTCATGTATTGAAGAGTGAATTT
*gsr*	2936	Forward: CCAGCTTAGGAATAACCAGCGATGG
		Reverse: GTCTTTTTAACCTCCTTGACCTGGGAGAAC
*gpx1*	2876	Forward: CGCTTCCAGACCATTGACATC
		Reverse: CGAGGTGGTATTTTCTGTAAGATCA
*gapdh*	2597	Forward: *TGCACCACCAACTGCTTAG*
		Reverse: *GATGCAGGGATGATGTTC*

**Table 3:** IC_50_ values of the tested OO extracts as calculated for the ABTS^·+^ and plasmid relaxation assays. The results are expressed as mean ± SEM (*n* = 3). OLE17 and OLE19 are HT-rich olive oils. OLE05 is a T-rich olive oil, and OLE20 and OLE50 are olive oils with an equal amount of HT and T. Means without a common letter are significantly different (*p* < 0.05). HT: hydroxytyrosol; T: tyrosol.

Extracts	ABTS^·+^ assay IC_50_ (*μ*g of extract)	Plasmid relaxation assay IC_50_ (*μ*g of extract)
OLE17	28.58 ± 0.193^a^	14 ± 0.866^a^
OLE19	20.75 ± 0.108^b^	22.5 ± 1.089^a^
OLE05	46.55 ± 0.460^c^	132.67 ± 1.027^b^
OLE20	35.68 ± 0.410^d^	74 ± 1.060^c^
OLE50	29.56 ± 0.283^a^	65.67 ± 1.649^c^

**Table 4:** The concentrations at which the tested OO extracts exhibited cytotoxicity assessed with the XTT assay and the range of the extract concentrations used for the evaluation of cell redox status. OLE17 and OLE19 are HT-rich olive oils. OLE05 is a T-rich olive oil, and OLE20 and OLE50 are olive oils with an equal amount of HT and T. HT: hydroxytyrosol; T: tyrosol. The cytotoxicity (%) at the cytotoxic concentration has been calculated compared with the control.

Extracts	Cytotoxic concentration (*μ*g of extract)	Cytotoxicity (%) at the cytotoxic concentration	Concentration range (*μ*g of extract)
OLE17	30.0	42	10.0-25.0
OLE19	30.0	55	5.0-20.0
OLE05	50.0	39	10.0-25.0
OLE20	50.0	42	10.0-25.0
OLE50	20.0	31	2.5-15.0

## Data Availability

The data used to support the findings of this study are included within the article.
